# Randomized controlled trial for selective preventive transdiagnostic intervention for adolescents at risk for emotional disorders

**DOI:** 10.1186/s13034-023-00616-9

**Published:** 2023-06-23

**Authors:** Manuel Vivas-Fernandez, Luis-Joaquin Garcia-Lopez, Jose A. Piqueras, Jose-Antonio Muela-Martinez, Josefa Canals-Sans, Lourdes Espinosa-Fernandez, David Jimenez-Vazquez, Maria del Mar Diaz-Castela, Paula Morales-Hidalgo, Maria Rivera, Jill Ehrenreich-May

**Affiliations:** 1 University of Jaen, Jaen, Spain; 2 Miguel Hernandez University, Elche, Spain; 3 Universitat Rovira I Virgili, Tortosa, Spain; 4 University of Miami, Miami, USA; 5 Division of Clinical Psychology, Department of Psychology, University of Jaen, Campus de las Lagunillas s/n, C-5., Jaen, Spain; 6 Universitat Oberta de Catalunya, Barcelona, Spain

**Keywords:** Adolescence, Anxiety, Depression, Randomized control trial, Selective prevention, Transdiagnostic

## Abstract

Significant evidence does exist on the effectiveness of transdiagnostic interventions to improve emotional problems in clinical populations, and their application as universal and indicated prevention programs. However, no randomized controlled trials (RCT) studying selective transdiagnostic prevention intervention have been published. This is the first known RCT to evaluate the efficacy/effectiveness of an evidence-based selective prevention transdiagnostic program for emotional problems in adolescents. The impact of three different interventions was evaluated: (1) PROCARE (Preventive transdiagnostic intervention for Adolescents at Risk for Emotional disorders), which is a group-based, abbreviated version of the Unified Protocol for Transdiagnostic Treatment of Emotional Disorders in Adolescents (UP-A), along with adding a booster session to reduce risk of onset of anxiety and depression, (2) PROCARE + , which includes the PROCARE protocol along with personalized add-on modules tailored to match adolescents’ risk factors, and (3) an active control condition (ACC) based on emotional psychoeducation. In total, 208 adolescents (48.5% girls) evidencing high risk and low protective factors were randomized and allocated to PROCARE, PROCARE + or ACC. Data from 153 adolescents who completed all assessments in the different phases of the study were analyzed. Self- and parent-reported measures were taken at baseline, as well as after the intervention, a 6 month follow-up was carried out, together with a 1 month follow-up after the booster session. Differences between conditions were significant on most of the outcome measures, with superior effect sizes for PROCARE + in the short and long term. Interventions were acceptable in terms of acceptability, with good satisfaction rates. Tailored targeted selective transdiagnostic interventions focused on mitigating risk factors and promoting protective factors in vulnerable adolescents are promising.

## Highlights

- Selective transdiagnostic preventive intervention with personalized add-on modules was effective in improving the emotional health and wellbeing of adolescents.
- Selective transdiagnostic prevention interventions reduced levels of emotional risk in adolescents and increased resilience.
- A 6-month follow-up booster session contributed to maintenance of effects for selective preventive intervention with add-on modules.

## Introduction

Emotional disorders are the leading causes of the global health-related burden, with depressive and anxiety disorders contributing the most to this burden [112]. Globally, approximately 117 million young people are affected by anxiety and/or depression [80]. Recently, the COVID-19 pandemic has exacerbated these problems, especially in young people. The worldwide prevalence and burden of depressive and anxiety disorders have increased massively. Data from 204 countries and territories demonstrate a 27.6% increase for major depressive disorders (an additional 53 million cases) and a 25.6% increase for anxiety disorders (an additional 76 million cases) [91]. Consistently, it is estimated that approximately another 260 million youth are at-risk for such concerns in the wake of the COVID-19 pandemic [43].

Adolescence is a sensitive window of opportunity to detect and intervene on emotional concerns, since more than half of such problems in adulthood have an age of onset before 14, with three quarters experiencing these concerns before the age of 24 [95]. The age range between 12 and 17 years constitutes a period of higher risk for anxiety and depression symptom onset [46] and such symptoms confer the greatest individual and social burden of all mental health difficulties [29, 108]. If left untreated, early onset anxiety and depression disorders are negatively related to social and family functioning, psychological distress, poor academic performance and increased suicidality [7, 10, 25, 41, 59, 87, 94, 103, 107].

Economic, social and personal costs of emotional problems among young people are extraordinarily high and therefore have been considered as priority conditions addressed in the World Health Organization (WHO) Mental Health Gap Action Programme (mhGAP; [111, 112]. Failure to address adolescent mental health can have broad negative implications both now and, in the future, limiting their opportunities to lead healthy and fulfilling lives as adults. Major depressive disorder carries an increased risk for adolescents, as it is associated with a seven-fold increased risk of suicide compared to adolescents without the disorder [104]. Although caring for adolescents at risk for anxiety and depression is of prime importance, only 20–30% of adolescents with clinically-significant emotional disorders access evidence-based interventions at that age and, even when they do access such treatments, drop-out rates are high [3, 57, 68, 69].

Potentially owing to the high comorbidity between anxiety and depression and higher order factors which may provide a common risk profile for anxiety and depression [6, 101], the *Unified Protocol for Transdiagnostic Treatment of Emotional Disorders in Adolescents* (UP-A; [27] appears to be a solid, evidence-based transdiagnostic approach for young people in clinical populations, and recently for universal prevention [28, 42, 45, 60, 101], with a B level of recommendation [35]. The transdiagnostic approach to Cognitive Behavioral Therapy (CBT) programs addresses common core mechanisms across emotional disorders (e.g., negative affectivity, stress, emotional avoidance) rather than specific-disorder prevention interventions [90]. Multiple studies support this approach because of the high rates of comorbidity between mood and anxiety disorders, the generally similar response to treatment between the disorders, shared neural activation patterns, and shared etiologic vulnerabilities [77, 109] The relevance of psychological interventions that are more appropriate for patients with comorbid psychiatric and medical conditions has been increasingly recognized through this unified approach [8].

Evidence-based, preventive interventions are an effective approach to support adolescents at risk for developing emotional problems before full symptoms evolve. Prevention can be aimed at bolstering resilience in the face of adversity by improving young people's ability to cope with difficult situations, ultimately preventing the later onset of more severe emotional problems [13, 52, 98, 105]. In particular, universal or indicated prevention programs have been developed to prevent depression and anxiety during adolescence, with positive findings regarding the reduction of emotional problems and risk for developing clinical disorders, although most studies report small effect sizes for such programs [15, 20, 21, 33, 34, 40, 54, 58, 64, 70, 71, 93, 97, 107]. In addition, review studies suggest that selective prevention programs have stronger effects than indicated or universal prevention, allowing a more cost-efficient, personalised intervention by targeting a specific group of individuals who are at higher risk, rather than offering population-wide interventions [45, 98]. Despite promising early evidence, only 20% of randomized controlled trials (RCT) studies on selective preventions include some active control condition (ACC) [11]. The use of waitlist control conditions (WLCs) in RCTs may also overestimate treatment effects and thus artificially inflate the effect sizes of prevention programs [1, 26, 31, 36, 74]. Further, it is suggested that the positive effects diminish over time [17]. In order to palliate this, the effect of booster sessions has been studied, concluding that booster sessions increase the effects of CBT training [44]. However, no research has been conducted to examine the impact of booster sessions in preventive transdiagnostic interventions.

It is worth noting that screening adolescents with putative risk factors (i.e., parental rearing, history of bullying, unhealthy habits, exposure to stress-related conditions) for emotional disorders is essential to improve their functioning and well-being [5, 65, 106] and to potentially prevent the development of more significant clinical disorders [16]. Nonetheless, no RCT has been conducted examining the impact of new personalized CBT, transdiagnostic preventive interventions to be implemented as add-on modules to the existing UP-A protocol. Add-on modules may be tailored to the risk factors identified and evidenced by at-risk adolescents under a personalized medicine approach.

To address this lack of well-founded and evidence-based selective prevention programs with a transdiagnostic focus for adolescents at risk for emotional disorders, this study aims: to provide data for acceptability, fidelity, and adherence to the three interventions; to evaluate differences in each of the three treatments in terms of emotional risk, resilience and quality of life related to physical, mental and social health as primary outcomes and emotional regulation skills, cognitive flexibility and anxiety and depression symptomatology as secondary outcomes; and to compare the three treatments to determine which group shows the greatest differences. In particular, RCT will be implemented through a 3-arm trial to examine the efficacy of a CBT transdiagnostic selective prevention intervention as an adaptation of UP-A to 8 sessions, conducted in telepsychology and group format, along with including add-on modules to target adolescents’ needs and particular risk factors named as PROCARE + , compared to the core intervention but without add-on modules (PROCARE) and an active control condition (ACC). A booster session is conducted 6 months after intervention in order to maintain the benefits over time. In summary, the present trial evaluated the efficacy of a new personalized CBT transdiagnostic intervention that was implemented as an add-on to an abbreviated version of evidence-based UP-A, including a booster session that was delivered online to mitigate the risk of anxiety and depression in vulnerable adolescents.

## Material and methods

### Design

The study follows a 3-arm RCT (Arm 1 = ACC; Arm 2 = PROCARE; Arm 3 = PROCARE +) in Spanish-speaking adolescent population. For the purpose of testing the efficacy and efficiency of these programs, we followed the Consolidated Standards of Reporting Trials (CONSORT: http://www.consort-statement.org) and the SPIRIT guidelines (Standard Protocol Items: Recommendations for Intervention Trials). The study was registered at the ClinicalTrial.gov database: Identifier: NCT04851366.

Primary and secondary outcome measures assessed risk of emotional problems, resilience, quality of life, emotional regulation skills, psychological flexibility, anxious-depressive symptomatology and additional risk and protective factors at posttest and at the 6 month follow-up. After follow-up, a booster session was implemented followed by a 1 month follow-up to evaluate the impact of booster sessions in maintenance of gains after the intervention. Both the assessments and the group intervention were conducted online using telepsychology (Google Meet) because of social distancing measures due to COVID-19 pandemic. PROCARE received Institutional Review Board (IRB) approval and followed the American Psychological Association (APA) standards and Guidelines for the Practice of Telepsychology [4]. All assessments were performed in an online format through a secure platform. This study was approved by the Bioethics Committee of the University of Jaen, ID: GEN-3461-aab8-41a3-85c2-ca28-5102-cdda-8d53.

### Participants

The screening included 1487 adolescents aged between 12 and 18 years (M = 14.32; SD = 1.759). Specifically, 887 self-reported their gender as female (59.7%), 583 as male (39.2%) and 17 as non-binary gender (1.1%). Self-reported information was obtained from 1211 parents or legal guardians about the emotional state of the adolescents (in those under 16 years of age). The ethnic composition of the sample included a 4.4% migrant population, in line with the Spanish census  [56].

The inclusion criteria for the RCT were: (1) having the informed consent of the adolescent and his or her guardian or legal custodian, (2) the technological means to attend the online sessions; (3) possible risk of emotional problems reported by the Spanish version of the emotional symptoms subscale of the Strengths and Difficulties Questionnaire (SDQ) in the Self-Reported or the Parent-Reported version [9, 76] (4) low or medium resilience reported by the *10-Item Connor-Davidson Resilience Scale* (CD-RISC-10; [18, 75] (5) low overall emotional symptomatology or scores below normative data for any of the subscales (depression, panic, social phobia, separation, generalized anxiety and obsessive compulsive disorder measured with the Revised Children’s Anxiety and Depression Scale (RCADS-30; [79, 89], (6) presence of at least one risk factor (social exclusion, stress-related situations, unhealthy lifestyle habits, parental-child interaction), (7) not receiving psychological or psychiatric treatment, (8) not presenting acute suicidality and (9) absence of neurodevelopmental disorders.

**Fig. 1:**
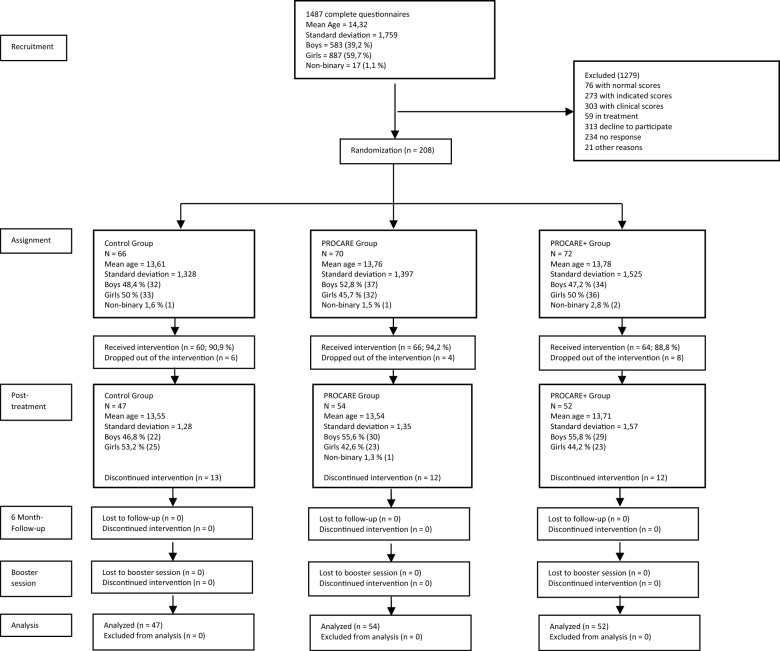
Consort flow diagram

In order to estimate sample size, G*Power was calculated to prove an effect size of at least d = 0.25 (Cohen’s *d*) with 80% power. The selected sample of the study consisted of 208 adolescents (48.5% girls, 49.6% boys and 1.9% non-binary gender), with a mean age of 13.71 (SD = 1.41; range = 12–18) and 192 parents or legal guardians. As can be seen in Fig. 1, the adolescents were randomly allocated into the three treatment conditions: ACC (n = 66), PROCARE (n = 70) and PROCARE + (n = 72). The distribution was homogeneous (see Table 1) and there was no interdependence relation between the experimental conditions and any of the sociodemographic variables tested (*p* > 0.05).

**Table 1:** Socio-demographic variables

	ACC *M* (*SD*)	PROCARE *M* (*SD*)	PROCARE + *M* (*SD*)	
N	47	54	52	*ns*
Age	13.55 (1.28)	13.54 (1.35)	13.71 (1.57)	*ns*
Gender				*ns*
Girls	25 (53.2%)	23 (42.6%)	23 (44.2%)	
Boys	22 (46.8%)	30 (55.6%)	29 (55.8%)	
Non-binary	0 (0%)	1 (1.3%)	0 (%)	
Nationality				*ns*
Spanish	42 (89.4%)	47 (87%)	48 (92.3%)	
Non-Spanish	5 (10.6%)	7 (13%)	4 (7.7%)	
Attendance (0–8)	7.62 (0.76)	7.70 (0.57)	7.62 (0.59)	*ns*

*M*: mean; *SD*: standard deviation; *ns*: non-significant (*p* >0 .05)

### Measures

The assessment of the emotional state of the adolescents, prior to the intervention and in subsequent evaluations, included the following instruments.

#### Primary outcome measures

*The Strengths and Difficulties Questionnaire* (SDQ; [47] (www.sdqinfo.org). It is a measure of emotional and behavioral difficulties in children and adolescents, translated into several languages, including Spanish. It consists of 25 items with Likert-type response format scored from 0 to 2 (‘‘not true’’, ‘‘somewhat true’’ and ‘‘certainly true’’ grouped into 5 subscales: emotional symptoms, conduct problems, hyperactivity/inattention, peer relationship problems and prosocial behavior. The self-reported version for adolescents (Self-Reported SDQ was used. For parents or legal guardians, only the 5 items of the emotional problems subscale of the parent version (Parent SDQ were used. Self-reported and parents or legal guardians version shown adequate psychometric properties and cut-off scores for screening purposes [9, 76] In this study, the reliability (Cronbach’s alpha; α value was 0.81 and 0.83 for self-reported and parent-reported versions, respectively.

*10-Item Connor-Davidson Resilience Scale* (CD-RISC-10; [18]. It is a reduction of the original [24]. It consists of 10 items with a Likert-type response format from 0 to 4 (‘‘not at all’’, ‘‘rarely’’, ‘‘sometimes’’, ‘‘often’’, and ‘‘almost always’’). The Spanish version was used for this study, which has shown good psychometric properties and is considered a reliable and valid instrument for measuring resilience [75]. In this study, Cronbach’s α was 0.92.

*KIDSCREEN-10 Index.* [86]. Questionnaire developed from the KIDSCREEN-27 which assesses the overall health-related quality of life of children and adolescents in relation to physical, mental and social health status. This instrument contains 10 items with a Likert-type response form ranging from 1 to 4 (‘‘not at all’’, ‘‘a little’’, ‘‘moderately’’, ‘‘a lot’’ and ‘‘very much’’). The psychometric properties are adequate [37, 85]. In this study, Cronbach’s α was 0.85.

#### Secondary outcome measures

*Difficulties in Emotion Regulation Scale (*DERS; [49]. The Spanish adaptation of [53] was applied, which has shown adequate psychometric properties in Spanish adolescents. It is a measure of emotional regulation, consisting of 36 items with a Likert-type response format ranging from 0 to 4 (‘‘Almost never’’, ‘‘sometimes’’, ‘‘half the time’’, ‘‘most of the time’’, ‘‘almost always’’) grouped into six dimensions: (1) non-acceptance of emotional responses, (2) difficulties in directing behavior towards goals when upset, (3) difficulties in controlling impulsive behaviors when upset, (4) effective emotional regulation strategies, (5) lack of emotional awareness and emotional clarity. In this study, Chronbach’ α was 0.82.

*Willingness and Action Measure for Children and Adolescents* (WAM-C/A; [50, 63]. The Spanish adaptation of Cobos-Sánchez et al. [22] was used, which has good psychometric properties. It is a measure of psychological flexibility which assesses the willingness to accept and be in contact with emotions, thoughts, feelings or emotional experiences generating discomfort (acceptance subscale), as well as the tendency to act in the direction of important values and life goals (action subscale). It has 14 items with Likert-type response format scored from 0 to 4 (‘‘not true at all’’, ‘‘a little true’’, ‘‘quite true’’, ‘‘true’’ and ‘‘very true’’). In this study, Chronbach’ α value was 0.82.

*The Revised Child Anxiety and Depression Scale, 30- item version,* RCADS-30 [89]. This is an adapted brief version of the original RCADS [19, 88]. It is a brief version consisting of 30 items with Likert-type responses scored from 0 to 3 (‘‘never’’, ‘‘sometimes’’, ‘‘often’’ and ‘‘always’’) which assess symptoms of anxiety and depression in children and adolescents. It consists of six subscales which are useful for screening adolescents in terms of high prevalence disorders: panic disorder (PD), social phobia (SP), separation anxiety disorder (SAD), generalized anxiety disorder (GAD), obsessive–compulsive disorder (OCD) and major depressive disorder (MDD). The total score was used as the primary outcome measure whereas subscales were secondary outcome measures. The RCADS-30 has excellent psychometric properties and cut-off points for Spanish populations [79]. In our study, the RCADS total score was found to have excellent mean reliability, with a mean alpha value of 0.89. Cronbach’s α for subscales ranged from 0.72 to 0.76, indicating acceptable reliability.

To identify putative risk factors evidenced by adolescents, the following measures were taken:

#### Social exclusion

*Cyberbullying and bullying scale* [38]. Victimization and cybervictimization scales were used for this study. The response format is Likert-type from 0 to 4 (‘‘never’’, ‘‘sometimes’’, ‘‘quite often’’ and ‘‘always”), indicating the frequency in which the participant has been (cyber) victimized during the last year. The psychometric properties of the instrument are good [39]. In this study, Cronbach’s α was 0.86. Additionally, the question ‘‘Have you ever felt discriminated against for any reason (for example, being part of the LGBTIQ + community, being a migrant, refugee, of another ethnicity, because of your religion or language)?’’ was added ad-hoc to evaluate risk of social exclusion.

#### Stress-related situations

As RCT was conducted during pandemic, situations were focused on Covid-19 stressors.

Fear of COVID-19 Scale (FCV-19S; [2]. The Spanish adaptation of Piqueras et al. [78] was employed. The scale consists of 7 items answered on a Likert scale from 1 to 5 (‘‘Strongly disagree’’, ‘‘disagree’’, ‘‘neither agree nor disagree’’, ‘‘agree’’ and ‘‘agree and strongly agree’’). In this study, it was proposed as a risk factor to score 19 or more on the scale or to have had a recent experience with COVID-19 by scoring ‘‘Yes’’ on item 8: ‘‘Is there a member of your family or a friend who has been infected by COVID-19?’’. The psychometric properties of the instrument are good for both international and Spanish samples [2, 78]. In this study, Cronbach’s α was 0.81.

#### Health and lifestyle habits

A short 9-question questionnaire was created ad-hoc to detect different problems related to health and lifestyle habits. To consider this risk factor, the presence of any of the following unhealthy habits was considered: regular consumption of substances (alcohol, tobacco or cannabis), daily exposure to screens greater than 4 hours, presence of sleep difficulties (difficulties in reconciling sleep, frequent awakenings during the night or tiredness in the mornings) or body dissatisfaction. In this study, Cronbach’s α was 0.73.

#### Parental-child interaction

*Structured Interview for the Assessment of Expressed Emotion: Child version* (E5cv [73]. Five-item structured interview with five response options, ranging from 1 to 5 (‘‘Never’’, ‘‘almost never’’, ‘‘sometimes’’, ‘‘almost always’’ and ‘‘always’’). Each item covers a dimension of Expressed Emotion: criticism, generalized hostility, hostile rejection, hopelessness, and self-sacrifice. To consider this risk factor, it was proposed to score ‘‘always’’ in one of the items. The scale showed good psychometric properties in Spanish-speaking adolescents with anxiety symptomatology [73]. In this study, Cronbach’s α was 0.86.

Additionally, participant satisfaction after treatment was assessed by the *Client Satisfaction Questionnaire* (CSQ-8; [62]. The CSQ-8 is a self-reported questionnaire assessing the general level of satisfaction with the service received. It is composed of 8 items which are scored on a scale, ranging from 1 to 4. The total score varies from 8 to 32, where a higher score indicates greater satisfaction with the service received. Good psychometric properties for the Spanish-speaking population have also been found by [102]. In this study, Chronbach’s α was 0.87.

### Procedure

This study was divided into several phases: screening, pre-test assessment and allocation to treatment conditions, 8-sessions of a 60 min length intervention, post-test assessment, a 6-month follow-up, a 90 min booster session and a 1 month follow-up after the booster session. Dissemination and recruitment in this study was carried out through secondary education centers, social media, radio and press release for general population. Dissemination reached society widely, thanks to the support of our strong nationwide external advisory board, formed by stakeholders such as governmental entities (The National Youth Institute), the third sector (The Youth Council of Spain), minorities (LGTBI + Young Group Federation), NGOs (Counselors National Association, COPOE) as well as end-users (Spanish Association for Mutual Assistance against Anxiety Disorders, AMTAES).

Informed consent was obtained from both legal guardians and the adolescents themselves (or limited to adolescents if their age was ≥ 16 years-old, according to Spanish law). During the screening phase, the SDQ (self-reported and parent-reported version), CD-RISC and RCADS were administered to identify the risk of developing emotional problems, low resilience and in order to rule out anxiety or depressive symptomatology. The assessment protocol was conducted through an online platform designed using the software application Limesurvey^©^, a tool which allows the secure development, publication and collection of data through online surveys. Assessors were blind to treatment allocation. A brief report with the results extracted from their scores was provided to the adolescents and their families. Those adolescents with anxiety or depressive symptomatology were referred to another prevention program for indicated population or to public mental health services. Participants evidencing at least one risk factor were eligible to enter the trial.

In total, 208 adolescents entered the trial, with the following distribution: ACC (n = 47), PROCARE (n = 54), or PROCARE + (n = 52) All participants were randomly assigned to the telehealth-delivered interventions and had no knowledge of which intervention they were receiving. 6 adolescents assigned to ACC declined to enter the trial claiming that it would interfere with their academic performance or because of perceived low usefulness. As for the PROCARE condition, 4 of adolescents were unable to commit to a treatment schedule and evidenced a low self-perception of risk factors. Moreover, 8 adolescents in the PROCARE + condition did not participate due to parents’ allegation of lack of time to attend parental add-on sessions (in those cases where parents were invited due to parent–child dysfunctional interactions as a risk factor) and reported conflict with academic activities. An intention-to-treat (ITT) analysis revealed no significant differences (*p* > 0.05) between the sample assigned to conditions and the one which definitely benefited from the experimental conditions. During treatment, 13, 12 and 12 adolescents dropped out of the sessions for ACC, PROCARE and PROCARE + , respectively. Consequently, sample size computed for data analysis consisted of 153 adolescents: ACC (n = 47), PROCARE (n = 54), or PROCARE + (n = 52). There were no differences between the completers and non-completers (*p* > 0.05).

Parents and adolescents were reassessed at post-treatment and follow-up period. According to the EU Clinical Trial Directive (2001/20/EC) and Regulation (536/2014), compensation to research participants was not a benefit and was not listed in the benefits section of the protocol. Recruitment techniques (e.g., advertising) did not focus on compensation as a means of enticing potential participants. Participants enrolled in the RCT and post treatment assessment did not receive any compensation. Only adolescents and parents participating in the booster session and follow-up assessments were eligible to be compensated for their time. The trial was planned according to internationally adopted guidelines (ICH-E6, E8 and E9), as well as pursuant to other guidelines, e.g., from the European Medicine Agency (EMA). PROCARE adhered to current data protection legislation (Regulation (EU) 2016/679).

### Experimental conditions

To encourage maximum fidelity to the protocol, prior to the start of the study, an online training with therapists was conducted within PROCARE, PROCARE + and ACC. High-level supervision of the UP-A techniques was performed by the developer of the intervention. All therapists passed all treatment competency verifications after training. Additional measures of protocol adherence and treatment integrity were developed during the RCT for both treatment conditions. Fidelity sheets were filled in by therapists after each session and were supervised by the team at the University of Miami in order to maintain maximum fidelity to the treatment content and manual instructions.

For all treatment conditions, sessions were group-based (6–8 adolescents), delivered via telepsychology (Google Meet) and ran by a therapist and a co-therapist certified by the University of Miami. The three conditions included a booster session (of a 90 min length) to maintain the effects of interventions over time. The youth booster session consisted of a 90 min session aimed at reviewing and refreshing participants' acquired skills during the course. Details of each line of treatment are provided below:

The PROCARE intervention was an abbreviated adaptation of the Unified Protocol for Transdiagnostic Treatment of Emotional Disorders in Adolescents (UP-A; [27]. The UP-A applies evidence-based CBT strategies for the treatment of emotional disorders such as emotion education, cognitive reappraisal, behavioral activation, and a range of exposure techniques,along with others such as motivation enhancement and mindfulness techniques. It is aimed to promote change through improvements in emotional reactivity and regulation skills, enhancing tolerance to distress associated with intense emotions and reducing or eliminating maladaptive emotional behaviors which reinforce the intensity of emotional distress in the long term. The present 8-sesion adaptation is only focused on adolescents and is aimed at developing their resilience using the core modules of the UP-A. Abbreviated versions of the following modules were delivered: (1) education about emotions and emotional behaviors, (2) introduction to emotion-focused behavioral experiments, (3) awareness of physical sensations, (4) flexible thinking, (5) emotional awareness, and (6) situation-based emotion exposures.

The PROCARE + intervention includes the entire content of the PROCARE program and additional modules are administered tailored according to the risk factor evidenced by the adolescents. The add-on youth module sessions were conducted in smaller groups of 5–6 participants and included three modules for adolescents and one module for parents. The three add-on youth modules targeted risk factors such as social exclusion, stress-related in relation to COVID-19 and healthy habits through one-hour length therapeutic sessions focused on providing adolescents with specific psychological tools such as communication skills, coping skills to manage stress, promotion of healthy lifestyle habits, critique of social influences and strategies to promote change. Those adolescents evidencing more than one risk factor (high risk) attended the consequent add-on modules. The add-on parental module sessions were designed to improve parent–child communication skills with a particular emphasis on reducing levels of parental expressed emotion. The add-on parental module consisted of 4 weekly 60 min group sessions (6–8 parents per group), delivered via telepsychology (Google Meet).

The ACC was an abbreviated 8 week adaptation of Utalk [61] preventive intervention for adolescents who are at risk for problems with social anxiety and/or depression. Utalk is based on emotional psychoeducation in group format, emphasizing discussion of thoughts, feelings and behaviors as parts of emotions such as fear, anger/frustration, happiness/excitement or sadness, and providing support around generally distressing events.

### Data analysis

Data were coded and analyzed with the IBM SPSS Statistics 28.0 [55]. First, the homogeneity of the sample was analyzed through Multivariate Analysis of Covariance (MANOVA) in the pre-test measurements, controlling the effect of age, gender, nationality and session attendance (as covariates). No interaction effects were found. MANOVAs were performed including conditions, sex and age as fixed factors in order to analyze possible indirect effects or interaction effects. No interaction effects between sex, age and conditions were found, so we proceeded with the next step. Second, MANOVAs were conducted at posttest, at the 6 month follow-up and at the 1 month follow-up after the booster session to examine the overall differences among the three experimental conditions once they had been tested to be equivalent in the pretest. In all cases, MANOVAs were adjusted for age and gender. After the MANOVAs, a variance analysis (ANOVA) of the post-test, follow-up, and post-booster session scores was conducted to assess the global effectiveness of the program. Third, between-group comparisons were undertaken. Thus, descriptive (means and typical deviations) and variance (ANOVA) analyses were carried out with each of the scores obtained in the experimental groups and the control group in the post-test and follow-ups. To control Type I error due to multiple comparisons, the Bonferroni adjustment was employed. Finally, within-group comparisons for each condition were calculated. Descriptive analyses (means and typical deviations) of the different experimental groups were conducted and the possible differences between pre-test and post-tests (post-intervention, follow-up and post-booster sessions) were analyzed using paired-samples Student's *t* test. Effect sizes were analyzed by means of Cohen’s *d* (typified mean difference) and eta-squared. The following recommendations were used for interpretation purposes: for parametric comparisons, Cohen’s *d*: small (a1) = 0.2, medium (a2) = 0.5, large (a3) = 0.8 was used; and for MANOVAS and for non-parametric tests, the eta-squared was applied: small (b1) = 0.01, medium (b2) = 0.06, large (b3) = 0.14 [23]. The relationship between treatments and improvements was assessed using a Pearson's Chi-Square Test of Association.

## Results

### Attendance, feasibility, fidelity and acceptability rates

The attendance of participants to sessions was high, with no differences among conditions, ACC (*M* = 7.62, *SD* = 0.76), PROCARE (*M* = 7.70, *SD* = 0.57) and PROCARE + (*M* = 7.62, *SD* = 0.59), *H*(2) = 1.38, *p* = 0.53. The fidelity of therapists to the treatment content and manuals was 98.7%, 97.1% and 97% for ACC, PROCARE and PROCARE + , respectively. Participation of adolescents was also high (0–16), ACC (*M* = 9.57, *SD* = 3.79), PROCARE (*M* = 10.75, *SD* = 3.41) and PROCARE + (*M* = 9.07, *SD* = 3.93), *F* = 2.49, *p* = 0.91. Fidelity sheets were filled in by therapists after each session and were supervised by the team at the University of Miami in order to maintain maximum fidelity to the treatment content and manual instructions. Good satisfaction levels were found, measured by Client Satisfaction Questionnaire (CSQ-8; range: 0–32), with no statistically differences in ACC (*M* = 27.93, *SD* = 3.12), PROCARE (*M* = 29.17, *SD* = 3.29) and PROCARE + (*M* = 29.26, *SD* = 3.54) conditions, *H*(2) = 0.36, *p* = 0.87. The degree of satisfaction of adolescents with the PROCARE + add-on youth modules was: partly satisfied (3%), satisfied (10.4%), very satisfied (57.2%) and totally satisfied (29.4%). Parental add-on module scored by parents was: 63.2% totally satisfied and 36.8% very satisfied.

### Between-group analyses

The effects of the interventions on the outcome variables (except for the inclusion of the RCADS total score, which is a sum of the included RCADS subscales scores) were examined using MANOVA adjusted for age and gender (see Table 2). Results revealed no statistically significant differences among the conditions at pretest (Wilks Lambda *Λ* = 0.44, *F* (2, 152) = 1.12, *p* = 0.11). Thus, a main effect of age, gender, or condition was not found at pretest. The results of the ANOVA in the baseline/pre-treatment phase suggest that there were no significant differences in any of the measures between the experimental groups and the ACC.

**Table 2:** *Global inter-group comparisons (ACC, PROCARE, PROCARE* +*)*

*Measures*	*Baseline/pre-treatment* *Mean (SD)*			*Post-treatment Mean (SD)*			*6-months Follow-Up Mean (SD)*			*Post-booster Mean (SD)*			*Effect Size (ε*^*2*^*)*		
	ACC	PROCARE	PROCARE +	ACC	PROCARE	PROCARE +	ACC	PROCARE	PROCARE +	ACC	PROCARE	PROCARE +	Post-treatment	6-months Follow-Up	Post-booster
*Primary outcome measures*															
Self-Report SDQ	2.68 (1.78)	3.06 (1.80)	2.88 (1.40)	2.57 (1.69)	2.39 (2.08)	1.63 (1.46)	2.94 (2.15)	2.41 (1.82)	1.92 (1.49)	2.89 (2.34)	1.98 (1.81)	1.52 (1.57)	0.05*_b1_	0.03*_b1_	0.07_b2_**
Parent SDQ	3.53 (2.43)	3.72 (2.37)	2.98 (2.01)	2.53 (2.20)	2.44 (1.69)	1.52 (1.63)	2.77 (2.02)	2.54 (1.69)	1.50 (1.63)	2.68 (1.92)	2.02 (1.56)	1.17 (1.42)	0.06**_b2_	0.1_b2_***	0.12_b2_***
CD-RISC	25.47 (5.53)	25.35 (5.32)	26.50 (5.50)	27.23 (6.55)	27.37 (6.96)	29.31 (6.23)	26.19 (5.49)	28.33 (7.63)	29.92 (6.64)	26.91 (6.34)	28.56 (5.76)	32.69 (5.76)		0.05_a1_*	0.15_b3_***
KIDSCREEN	36.19 (5.29)	35.72 (5.14)	36.46 (5.30)	36.89 (6.01)	37.83 (5.78)	38.38 (5.85)	35.53 (6.57)	38.11 (5.83)	38.48 (5.72)	36.13 (5.97)	38.48 (6.06)	40.48 (5.66)		0.04_a1_*	0.08_a2_**
*Secondary outcome measures*															
DERS	79.55 (24.73)	78.89 (17.91)	78.33 (20.32)	80.96 (24.26)	77.07 (21.83)	71.65 (18.21)	77.60 (20.49)	72.30 (20.45)	68.31 (17.48)	76.87 (21.36)	68.74 (21.55)	64.50 (18.24)			0.06_a2_**
WAM	39.87 (9.10)	38.69 (9.35)	39.90 (8.33)	40.64 (12.02)	44.13 (11.19)	44.23 (9.90)	41.72 (10.89)	45.02 (11.67)	42.54 (9.82)	42.47 (11.82)	43.72 (11.38)	45.87 (9.63)			
RCADS (Total)	19.55 (8.01)	22.24 (8.52)	21.54 (8.94)	21.06 (9.05)	19.41 (11.02)	17.50 (10.72)	21.55 (11.09)	19.67 (11.60)	17.52 (9.60)	21.00 (12.75)	16.19 (10.11)	14.44 (9.76)			0.04_b1_*
RCADS (GAD)	6.04 (2.59)	6.20 (2.62)	7.13 (2.51)	5.72 (2.82)	5.67 (3.30)	5.88 (3.28)	6.19 (3.34)	5.87 (3.41)	5.56 (2.87)	5.70 (3.70)	4.78 (2.65)	4.56 (3.11)			
RCADS (SoP)	4.66 (2.72)	6.02 (3.49)	5.27 (3.16)	5.00 (3.21)	4.96 (3.31)	3.96 (2.93)	5.13 (3.70)	5.17 (3.25)	4.23 (2.88)	5.17 (3.38)	4.43 (2.99)	3.38 (2.75)			0.05_a1_*
RCADS (PD)	1.34 (1.61)	1.26 (1.26)	1.38 (1.62)	1.89 (2.00)	1.48 (1.79)	1.23 (2.11)	1.85 (2.12)	1.44 (1.88)	1.06 (1.42)	1.91 (2.77)	1.02 (2.00)	0.63 (0.97)			0.04_b1_*
RCADS (MDD)	3.45 (2.19)	3.98 (2.12)	3.15 (1.93)	4.09 (2.38)	3.09 (2.27)	2.62 (2.13)	3.87 (2.79)	3.26 (2.47)	2.75 (2.09)	3.96 (2.57)	2.91 (2.17)	2.46 (2.14)	0.07_b2_**		0.06_b2_**
RCADS (SAD)	1.32 (1.73)	1.57 (1.81)	1.56 (2.52)	1.26 (1.78)	1.35 (1.46)	0.77 (1.28)	1.28 (2.02)	1.11 (1.76)	0.96 (1.66)	1.36 (2.24)	0.83 (1.54)	0.65 (1.40)	0.04_b1_*		
RCADS (OCD)	2.74 (1.92)	3.20 (2.40)	3.04 (2.32)	3.36 (2.27)	2.93 (2.24)	3.00 (2.67)	3.19 (2.24)	2.70 (2.21)	2.50 (2.37)	2.89 (2.40)	2.17 (2.06)	2.08 (2.19)			

Self-Report SDQ: *The Strengths and Difficulties Questionnaire (Adolescents. Emotional Problems Subscale)*; Parent SDQ: *The Strengths and Difficulties Questionnaire (Parents) Emotional Problems Subscale;* CD-RISC: *10-Item Connor-Davidson Resilience*; KIDSCREEN: *KIDSCREEN-10 Index;* DERS: Difficulties in Emotion Regulation Scale; WAM: Willingness & Action Measure for Children and Adolescents; RCADS (Total): Revised Children’s Anxiety and Depression Scale. Total score; RCADS (GAD): RCADS Generalized Anxiety Disorder subscale; RCADS (SoP): RCADS Social Phobia subscale; RCADS (PD): RCADS Panic Disorder subscale; RCADS (MDD): RCADS Major Depressive Disorder subscale; RCADS (SAD): RCADS Separation Anxiety Disorder subscale; RCADS (OCD): RCADS Obsessive Compulsive Disorder subscale

Effect size: ε2(parametric): small (a1) =0 .03, medium (a2) = 0,06

Effect size: ε2(non-parametric): small (b1) = 0.03, medium (b2) =0 .06, large (b3) = 0.14

^*^p ≤ 0.05

^**^p ≤0 .01

^***^p ≤ 0.001

#### Primary outcomes

At post-treatment, the MANOVA revealed significant differences among the conditions (Wilks Lambda *Λ* = 0.48, *F* (2, 152) = 0.98, *p* = 0.98), with a medium-to-large effect size (*η*^*2*^ = 0.12). Significant differences in primary outcomes such as the self-reported and parent-reported SDQ scales were found with small to medium effect sizes. Differences in mood and separation anxiety symptomatology with small to medium effect sizes were observed. By the 6 month follow-up, no significant differences were found (Wilks Lambda *Λ* = 0.55, *F* (2, 152) = 0.81, *p* = 0.81). Data showed significant differences in all primary outcomes, although small effect sizes were calculated, except for the level of emotional risk reported by parents (large effect size). At the 1 month follow-up after the booster session, significant differences were found (Wilks Lambda *Λ* = 0.57, *F* (2, 152) = 0.83, *p* = 0.75), with a large effect size (*η*^*2*^ = 0.14). Findings revealed that all primary outcome measures were significant, with medium to large effect sizes.

While the level of emotional risk was the only primary outcome to be significant at posttest, all primary outcome measures were statistically significant 6 month after the intervention and 1 month after the booster session. There was a unique pattern for both Self-reported and Parent-reported SDQ emotional subscale: significant differences were observed at all assessment times. Increases of resilience and quality of life were evident after the 6 month follow-up, with additional increase after the booster session, with larger effect sizes. Indeed, effect sizes were consistently larger after the booster session across measures.

Post-hoc comparisons between ACC and PROCARE conditions (see Table 3) showed no significant differences.

**Table 3:** *Post-hoc comparisons*

Measures	ACC vs PROCARE (PR) effect size (Cohen's *d*/Pearson's *r*)			ACC vs PROCARE + (PR +) effect size (Cohen's *d*/Pearson's *r*)			PROCARE vs PROCARE + (PR +) effect size (Cohen's *d*/ Pearson’s *r*)		
	Post-treatment	6 months follow-up	Post-booster	Post-treatment	6-months Follow-Up	Post-booster	Post-treatment	6 months Follow-Up	Post-booster
*Primary Outcome Measures*									
Self-Report SDQ				ACC < PR + 0,28_b1_**		ACC < PR + 0,31_b2_***			
Parent SDQ				ACC < PR + 0,24_b1_*	ACC < PR + 0,32_b2_***	ACC < PR + 0,42_b2_***	PR < PR + 0,28_b1_**	PR < PR + 0,29_b1_**	
CD-RISC						ACC < PR + 0,44_b2_***			PR < PR + 0,35_b1_***
KIDSCREEN				ACC < PR + 0,25_a1_***					
*Secondary Outcome Measures*									
DERS						ACC < PR + 0,38_a1_**			
WAM									
RCADS (Total)						ACC < PR + 0,25_b1_**			
RCADS (GAD)									
RCADS (SoP)						ACC < PR + 0,58_a2_**			
RCADS (PD)									
RCADS (MDD)				ACC < PR + 0,31_b2_**		ACC < PR + 0,30_b2_**			
RCADS (SAD)							PR < PR + 0,24_b1_**		
RCADS (OCD)									

Self-Report SDQ: *The Strengths and Difficulties Questionnaire (Adolescents. Emotional Problems Subscale)*; Parent SDQ: *The Strengths and Difficulties Questionnaire (Parents) Emotional Problems Subscale;* CD-RISC: *10-Item Connor-Davidson Resilience*; KIDSCREEN: *KIDSCREEN-10 Index;* DERS: Difficulties in Emotion Regulation Scale; WAM: Willingness & Action Measure for Children and Adolescents; RCADS (Total): Revised Children’s Anxiety and Depression Scale. Total score; RCADS (GAD): RCADS Generalized Anxiety Disorder subscale; RCADS (SoP): RCADS Social Phobia subscale; RCADS (PD): RCADS Panic Disorder subscale; RCADS (MDD): RCADS Major Depressive Disorder subscale; RCADS (SAD): RCADS Separation Anxiety Disorder subscale; RCADS (OCD): RCADS Obsessive Compulsive Disorder subscale

Effect size: Cohen’s *d*: small (a1) = 0.2, medium (a2) = 0.5

Effect size: Pearson’s *r* (non-parametric): small (b1) = 0.1, medium (b2) = 0.3

^*^p ≤ 0.016

^**^p ≤0 ,01

^***^p ≤ 0.001

Post-hoc comparisons between ACC and PROCARE + found that the latter evidenced significant improvements in most primary outcome measures, especially in post-treatment and 1 month after the booster session with small-to-medium and medium effect sizes in all post-treatment evaluation times.

Comparison between PROCARE and PROCARE + revealed that PROCARE + was significantly superior in the reduction of level of emotional risk reported by parents at all assessment stages with small effect sizes. Participants in the PROCARE + condition significantly improved their resilience levels but only after the booster session with a small effect size.

#### Secondary outcomes

At post-treatment, significant differences were found in major depression and separation anxiety subscales of the RCADS, with medium and small effect sizes respectively. At 6 month follow-up, the data showed no significant differences in the secondary outcome measures. At the 1 month follow-up after the booster session, findings indicated significant differences in a larger number of secondary outcomes covering emotion regulation and anxiety and mood symptomatology, with small and medium effect sizes.

Overall, general anxiety and depressive, social phobia and panic symptomatology were only significant after the booster session. Further, differences for depressive symptomatology after intervention were shown after intervention and after a booster session, with medium effect sizes. There were significant differences on separation anxiety but limited to the posttest.

There was no significant differences in post-hoc comparisons between ACC and PROCARE conditions (see Table 3).

Post-hoc comparisons between ACC and PROCARE + found that emotion regulation was significantly better for PROCARE + at 1 month follow-up after the booster session, with medium effect sizes. In addition, PROCARE + exhibited significant reduction in RCADS mood symptomatology at post-treatment and after the booster session, with small and medium effect sizes. There was a reduction in mood symptomatology at posttreatment and after the booster session (not at the 6 month follow-up), while overall anxiety and mood symptoms, and specific social phobia was evident only after the booster session.

Comparison between PROCARE and PROCARE + revealed that PROCARE + was significantly superior in the reduction of level of symptomatology for separation anxiety, witch small effect size.

### Within-group analyses

#### Primary outcomes

Within-group analysis for the ACC (see Table 4) revealed significant differences between pretreatment and posttreatment and follow-ups limited to the the level of emotional risk reported by parents with medium effect size. In addition, resilience improved between follow-up and after the booster session with a small effect size.

**Table 4:** Within-group comparisons

Measures		Baseline/pre-treatment Mean (*SD*)	Post-treatment Mean (*SD*)	6 month F-U Mean (*SD*)	Post-booster Mean (*SD*)	Effect Size (Cohen's *d*/ Pearson's *r*)		
						Pre-post-treatment	Post-treatment-6-months Follow-Up	6 months follow-up- post-booster
*Primary Outcome Measures*								
Self-Report SDQ	ACC	2.68 (1.78)	2.57 (1.69)	2.94 (2.15)	2.89 (2.34)			
	PROCARE	3.06 (1.80)	2.39 (2.08)	2.41 (1.82)	1.98 (1.81)	0.34_b2_***		
	PROCARE +	2.88 (1.40)	1.63 (1.46)	1.92 (1.49)	1.52 (1.57)	0.65_b3_***		0.34_b2_**
Parent SDQ	ACC	3.53 (2.43)	2.53 (2.20)	2.77 (2.02)	2.68 (1.92)	0.49_b2_***		
	PROCARE	3.72 (2.37)	2.44 (1.69)	2.54 (1.69)	2.02 (1.56)	0.49_b2_***		0.36_b2_***
	PROCARE +	2.98 (2.01)	1.52 (1.63)	1.50 (1.63)	1.17 (1.42)	0.61_b3_***		0.30_b2_*
CD-RISC	ACC	25.47 (5.53)	27.23 (6.55)	26.19 (5.49)	26.91 (6.34)			0.16_b1_*
	PROCARE	25.35 (5.32)	27.37 (6.96)	28.33 (7.63)	28.56 (5.76)	0.29_a1_***		
	PROCARE +	26.50 (5.50)	29.31 (6.23)	29.92 (6.64)	32.69 (5.76)	0.48_b2_***		0.65_a2_***
KIDSCREEN	ACC	36.19 (5.29)	36.89 (6.01)	35.53 (6.57)	36.13 (5.97)			
	PROCARE	35.72 (5.14)	37.83 (5.78)	38.11 (5.83)	38.48 (6.06)	0.33_a1_*		
	PROCARE +	36.46 (5.30)	38.38 (5.85)	38.48 (5.72)	40.48 (5.66)	0.37_b2_**		0.46_a1_***
*Secondary outcome measures*								
DERS	ACC	79.55 (24.73)	80.96 (24.26)	77.60 (20.49)	76.87 (21.36)			
	PROCARE	78.89 (17.91)	77.07 (21.83)	72.30 (20.45)	68.74 (21.55)			0.27_a1_*
	PROCARE +	78.33 (20.32)	71.65 (18.21)	68.31 (17.48)	64.50 (18.24)	0.37_a1_**		0.35_a1_**
WAM	ACC	39.87 (9.10)	40.64 (12.02)	41.72 (10.89)	42.47 (11.82)			
	PROCARE	38.69 (9.35)	44.13 (11.19)	45.02 (11.67)	43.72 (11.38)	0.67_a2_***		
	PROCARE +	39.90 (8.33)	44.23 (9.90)	42.54 (9.82)	45.87 (9.63)	0.38_b2_**		0.44_a1_***
RCADS (Total)	ACC	19.55 (8.01)	21.06 (9.05)	21.55 (11.09)	21.00 (12.75)			
	PROCARE	22.24 (8.52)	19.41 (11.02)	19.67 (11.60)	16.19 (10.11)			0.41_a1_*
	PROCARE +	21.54 (8.94)	17.50 (10.72)	17.52 (9.60)	14.44 (9.76)	0.45_b2_***		0.50_a2_***
RCADS (GAD)	ACC	6.04 (2.59)	5.72 (2.82)	6.19 (3.34)	5.70 (3.70)			
	PROCARE	6.20 (2.62)	5.67 (3.30)	5.87 (3.41)	4.78 (2.65)			0.38_b2_***
	PROCARE +	7.13 (2.51)	5.88 (3.28)	5.56 (2.87)	4.56 (3.11)	0.37_a1_**		0.42_a1_***
RCADS (SoP)	ACC	4.66 (2.72)	5.00 (3.21)	5.13 (3.70)	5.17 (3.38)			
	PROCARE	6.02 (3.49)	4.96 (3.31)	5.17 (3.25)	4.43 (2.99)			
	PROCARE +	5.27 (3.16)	3.96 (2.93)	4.23 (2.88)	3.38 (2.75)	0.39_b2_**		0.41_b2_***
RCADS (PD)	ACC	1.34 (1.61)	1.89 (2.00)	1.85 (2.12)	1.91 (2.77)			
	PROCARE	1.26 (1.26)	1.48 (1.79)	1.44 (1.88)	1.02 (2.00)			0.29_b1_*
	PROCARE +	1.38 (1.62)	1.23 (2.11)	1.06 (1.42)	0.63 (0.97)			0.36_b2_**
RCADS (MDD)	ACC	3.45 (2.19)	4.09 (2.38)	3.87 (2.79)	3.96 (2.57)	0.31_b2_*		
	PROCARE	3.98 (2.12)	3.09 (2.27)	3.26 (2.47)	2.91 (2.17)	0.39_b3_**		
	PROCARE +	3.15 (1.93)	2.62 (2.13)	2.75 (2.09)	2.46 (2.14)			
RCADS (SAD)	ACC	1.32 (1.73)	1.26 (1.78)	1.28 (2.02)	1.36 (2.24)			
	PROCARE	1.57 (1.81)	1.35 (1.46)	1.11 (1.76)	0.83 (1.54)			0.28_b1_*
	PROCARE +	1.56 (2.52)	0.77 (1.28)	0.96 (1.66)	0.65 (1.40)	0.44_b2_***		
RCADS (OCD)	ACC	2.74 (1.92)	3.36 (2.27)	3.19 (2.24)	2.89 (2.40)			
	PROCARE	3.20 (2.40)	2.93 (2.24)	2.70 (2.21)	2.17 (2.06)			0.35_b1_**
	PROCARE +	3.04 (2.32)	3.00 (2.67)	2.50 (2.37)	2.08 (2.19)			0.30_b2_*

*Note*: Self-Report SDQ: the strengths and difficulties questionnaire (adolescents. emotional problems subscale); parent sdq: the strengths and difficulties questionnaire (parents) emotional problems subscale; CD-RISC: 10-Item Connor-Davidson Resilience; KIDSCREEN: KIDSCREEN-10 Index; DERS: Difficulties in Emotion Regulation Scale; WAM: Willingness & Action Measure for Children and Adolescents; RCADS (Total): Revised Children’s Anxiety and Depression Scale. Total score; RCADS (GAD): RCADS Generalized Anxiety Disorder subscale; RCADS (SoP): RCADS Social Phobia subscale; RCADS (PD): RCADS Panic Disorder subscale; RCADS (MDD): RCADS Major Depressive Disorder subscale; RCADS (SAD): RCADS Separation Anxiety Disorder subscale; RCADS (OCD): RCADS Obsessive Compulsive Disorder subscale

Effect size: Cohen’s *d* small (a1) = 0.2. medium (a2) = 0.5

Effect size: Pearson’s *r* (non-parametric): small (b1) = 0.1. medium (b2) = 0.3. large (b3) = 0.5

^*^p ≤ 0.05.

**p ≤ 0.01

***p ≤ 0.001

Within-group analysis for PROCARE showed significant differences between pretreatment and posttreatment in all primary outcome measures. Effect sizes ranged from small to medium effect sizes. Statistically differences between 6 month follow-up and 1 month follow-up after a booster session were limited to the emotional risk perceived by parents with medium effect sizes.

Within-group analysis for the PROCARE + condition revealed differences between pretreatment and posttreatment in all primary outcome measures. Effect sizes ranged from medium to large. Statistical differences between the 6 month follow-up and the 1 month follow-up after the booster session included all primary outcome measures. Effect sizes ranged from small to medium effect sizes.

#### Secondary outcomes

Within-group analysis for the ACC revealed significant differences between pretreatment and posttreatment in mood symptomatology with a medium effect size.

Within-group analysis for PROCARE in psychological flexibility and depression symptomatology showed significant differences between pretreatment and posttreatment. Effect sizes ranged from medium to large effect sizes. Statistically differences between 6 month follow-up and 1 month follow-up after a booster session were found in emotion regulation and in anxiety and mood symptomatology in some variables, from small to medium effect sizes.

Within-group analysis for the PROCARE + condition revealed differences between pretreatment and posttreatment and follow-ups in most secondary outcome measures. Effect sizes ranged from small to medium. Statistical differences between the 6 month follow-up and the 1 month follow-up after the booster session included most of secondary measures except for mood and separation anxiety symptomatology. Most effect sizes ranged from small to medium effect sizes.

### Clinical significance

ACC group, consisting of 47 patients, showed improvement in 59.5% of cases after treatment, while 23.4% remained stable, and 17.1% worsened. PROCARE group, consisting of 54 patients, showed improvement in 75.9% of cases, while 16.6% remained stable, and 7.5% worsened. PROCARE + group, consisting of 52 patients, showed improvement in 82.7% of cases, while 13.5% remained stable, and 3.8% worsened. The relationship between treatments and improvements was assessed using a Pearson's Chi-Square Test of Association.

The results when compared ACC and PROCARE showed there to be no significant differences, *X*^2^ (2, N = 101) = 3.51, *p* = 0.17. The results when compared ACC and PROCARE + showed there is significant differences, *X*^2^ (2, N = 99) = 7.42, *p* = 0.02. There were marginally significant differences between PROCARE and PROCARE + , *X*^2^ (2, N = 106) = 0.92, *p* = 0.62.

## Discussion

This study was aimed at examining the efficacy of three selective preventive interventions in adolescents using a RCT. Overall, there were significant differences at post-test between conditions but limited to the level of emotional risk and anxiety symptomatology with small to medium effect sizes. This is consistent with findings from meta-analytic reviews suggesting that depression and anxiety prevention programs have small-to-medium effects on emotional health and wellbeing of adolescents [84]. However, all primary outcome measures were statistically significant across all conditions after the 6 month follow-up and particularly, after the booster session, mostly with medium-to-large effect sizes. Greater improvements obtained 6 months after the intervention and the booster session contrast with the absence of long-term effect evidenced by other prevention CBT trials [66, 84]. Likewise, review studies which focus on preventive interventions for anxiety and/or depression found that positive effects at short-term tend to decrease over time [98]. It has been argued that booster sessions may play a role to maintain the effects of interventions or reduce the likelihood of symptoms relapse over time, which could explain the desirable findings at follow-ups [84, 98]. Thus, selective intervention programs for at-risk adolescents can produce small to medium beneficial effects, bearing follow-up assessments and the booster session, in line with recommendations by [44]. Finally, this study is in line with other previous studies focused on the transdiagnostic approach, from which protocols are being developed with very promising results, targeting the treatment to the majority of problems experienced by adolescents rather than targeting symptoms of specific disorders [92, 110].

In particular, PROCARE has not shown significant differences compared to ACC when the groups are compared. Unlike PROCARE, PROCARE + showed statistical differences in all primary outcomes at follow-up with medium-to-large effect sizes and impacted in a greater number of secondary outcome measures. This suggests the importance of add-on modules tailored to the adolescents’ needs to reduce the level of emotional risk as reported by parents across all assessment times. Differences in resilience were only found in PROCARE + after the booster session, with medium effect size. As interventions were implemented during COVID-19 pandemic, data suggest that the regular practice of skills taught not only during core intervention but also during add-on modules to tackle specific risk factors, could help them building resilience during periods of increased stress such as the pandemic and potentially, during other traumatic events. Given that he COVID-19 pandemic has had a major impact on the emotional health, particularly in those adolescents who were at risk of developing psychopathology, the distinctive contribution of PROCARE + could have enhanced the emotional resilience [14, 32, 51, 81, 83, 91].

Within-group analyses also revealed differences in the profile of treatment gains for each condition. The ACC participants consistently evidenced differences between pretreatment and posttreatment and follow-up only in their level of emotional risk reported by parents, mood symptomatology and psychological flexibility. Positive findings are consistent with previous open trial study conducted by La Greca et al. [61]. However, PROCARE produced significant differences in all primary and secondary outcome measures, except for panic symptomatology, mostly with medium effect sizes. Differences between 1-month follow-up after the booster session and posttest or 6 month-follow-up were also found in many of the secondary measures, mostly with small effect sizes. Finally, PROCARE + exhibited significant differences between pretest and assessment stages, mostly with medium and large effect sizes. A greater number of differences were observed between the booster follow-up and the posttest or 6 month intervention, including large effect sizes. In addition, treatment gains (those who improved or maintained their status) were higher in that condition. Taking all of these data together, the three conditions evidenced positive impact on adolescents’ wellbeing but a larger number of differences and effect sizes were detected for PROCARE + . An additional finding is that SDQ emotional subscale (self-reported and/or parent-reported version) was particularly sensitive to treatment outcome for selective preventive purposes. This is in line with studies revealing that the SDQ was sensitive to treatment effects in clinical populations [48, 99].

Participants also reported high acceptability of all three interventions, including attendance to at least 95% of sessions, participation ranging between 57 to 67%, and excellent satisfaction rates being of 87–92%. These data are aligned with ACC findings by Utalk’s authors [61]. In case of PROCARE + , 86% of adolescents reported a very good or excellent satisfaction level for add-on youth modules and 100% of parents scored to be totally or very satisfied with the parental add-on module. This points out the excellent acceptability of PROCARE + add-on modules. In addition to the effectiveness of interventions, findings are aligned with recommendations proposed by some authors when implementing an evidence-based treatment into practice in terms of acceptability, appropriateness, feasibility, and fidelity [72, 82]. The study found that PROCARE and PROCARE + treatments resulted in higher rates of improvement compared to ACC group, being PROCARE + the group that showed the highest improvement rates, with 82.7% of cases showing improvement, being this rate of improvement higher than the average found in the literature [67].

### Limitations

First, a comprehensive health economic evaluation for the implementation of new interventions in healthcare and school settings is lacking. Estimation of potential good return on investment could support the implementation of selective, preventative interventions such as PROCARE + in different settings worldwide. Other limitations should be noted. Although a large number of participants reported high satisfaction levels, a few of them reported their preference to attend sessions in person. Future studies should investigate the costs and the cost-effectiveness of prevention programs and whether PROCARE + is equally beneficial to regular face-to-face therapy. Effects of the treatments were assessed at 6-month of intervention and one month after the booster session. Meta-analytic reviews have found that the benefits of preventive interventions tend to diminish at follow-ups [54, 100], so it is imperative to analyze the potential of these selective preventive interventions over the longer term. Finally, drop-out rates are consistent with previous meta-analysis that shows that the dropout rate in child and adolescent population is high [110]. The drop-outs reasons in this study should be examined to be addressed in future trials in order to reduce them. As a result of these drop-outs, the sample size has decreased at each stage of the study, which may have affected the statistical strength of the data, so future studies with larger samples will be necessary.

## Conclusions

For the first time, a selective transdiagnostic preventive intervention was tested, with promising results. PROCARE + was provided as an add-on to PROCARE, i.e., patients had access to an enhanced version of PROCARE by including add-on modules tailored to the risk factors identified and evidenced by participants. PROCARE + was superior to ACC in preventing emotional problems in at-risk adolescents. Overall, effect sizes were consistently larger across all conditions after the booster session, which suggests a positive impact of booster sessions on emotional health and wellbeing of adolescents. Furthermore, add-on modules seem to play a particular role in the increase of resilience.

## Data Availability

Data sets generated and/or analyzed during this study are not publicly available due to organizational limitations, but are available from the corresponding author upon reasonable request.
